# Role of the Sympathetic Nervous System in Carbon Tetrachloride-Induced Hepatotoxicity and Systemic Inflammation

**DOI:** 10.1371/journal.pone.0121365

**Published:** 2015-03-23

**Authors:** Jung-Chun Lin, Yi-Jen Peng, Shih-Yu Wang, Ton-Ho Young, Donald M. Salter, Herng-Sheng Lee

**Affiliations:** 1 Graduate Institute of Medical Sciences, National Defense Medical Center, Taipei, Taiwan, R.O.C; 2 Division of Gastroenterology and Hepatology, Department of Internal Medicine, Tri-Service General Hospital, National Defense Medical Center, Taipei, Taiwan, R.O.C; 3 Department of Pathology, Tri-Service General Hospital, National Defense Medical Center, Taipei, Taiwan, R.O.C; 4 Department of Pathology and Laboratory Medicine, Kaohsiung Veterans General Hospital, Kaohsiung, Taiwan, R.O.C; 5 Division of Gastroenterology, Department of Internal Medicine, Cardinal Tien Hospital, Fu Jen Catholic University, Taipei 231, Taiwan, R.O.C; 6 Center for Molecular Medicine, MRC IGMM, University of Edinburgh, Edinburgh, Scotland, United Kingdom; French National Centre for Scientific Research, FRANCE

## Abstract

Carbon tetrachloride (CCl_4_) is widely used as an animal model of hepatotoxicity and the mechanisms have been arduously studied, however, the contribution of the sympathetic nervous system (SNS) in CCl_4_-induced acute hepatotoxicity remains controversial. It is also known that either CCl_4_ or SNS can affect systemic inflammatory responses. The aim of this study was to establish the effect of chemical sympathectomy with 6-hydroxydopamine (6-OHDA) in a mouse model of CCl_4_-induced acute hepatotoxicity and systemic inflammatory response. Mice exposed to CCl_4_ or vehicle were pretreated with 6-OHDA or saline. The serum levels of aminotransferases and alkaline phosphatase in the CCl_4_-poisoning mice with sympathetic denervation were significantly lower than those without sympathetic denervation. With sympathetic denervation, hepatocellular necrosis and fat infiltration induced by CCl_4_ were greatly decreased. Sympathetic denervation significantly attenuated CCl_4_-induced lipid peroxidation in liver and serum. Acute CCl_4_ intoxication showed increased expression of inflammatory cytokines/chemokines [eotaxin-2/CCL24, Fas ligand, interleukin (IL)-1α, IL-6, IL-12p40p70, monocyte chemoattractant protein-1 (MCP-1/CCL2), and tumor necrosis factor-α (TNF-α)], as well as decreased expression of granulocyte colony-stimulating factor and keratinocyte-derived chemokine. The overexpressed levels of IL-1α, IL-6, IL-12p40p70, MCP-1/CCL2, and TNF-α were attenuated by sympathetic denervation. Pretreatment with dexamethasone significantly reduced CCl_4_-induced hepatic injury. Collectively, this study demonstrates that the SNS plays an important role in CCl_4_-induced acute hepatotoxicity and systemic inflammation and the effect may be connected with chemical- or drug-induced hepatotoxicity and circulating immune response.

## Introduction

Carbon tetrachloride (CCl_4_) is a chlorinated hydrocarbon that has widespread use in various industries as a solvent and in medicine as a vermifuge. It is found in low levels in ambient air and water [1,2]. Acute exposure to high levels of CCl_4_ vapor results in central nervous system depression and even coma. Lower levels of exposure leads to renal and especially hepatic toxicity [3,4]. Indeed CCl_4_ intoxication in the mouse is a widely used experimental model for the study of hepatotoxicity [1,5–7].

The mechanisms of CCl_4_ toxicity are recognized. CCl_4_ is mainly activated by cytochrome P450 2E1 to form the trichloromethyl peroxy radical, which initiates lipid peroxidation by pulling out a hydrogen atom in the vicinity of a polyunsaturated fatty acid double bond [1,2,8]. After propagation of the peroxidation process, lipids are finally degraded in small molecules such as malondialdehyde (MDA), a highly reactive aldehyde that can damage the plasma membranes [9]. The end result inactivates calcium pump activity with calcium influx. All these alterations eventually lead to liver cell death accompanied by the release into the blood of intrahepatic enzymes [1,3]. However, the mechanisms of CCl_4_-induced steatosis remain speculative [10].

A close relationship between the hepatic sympathetic nerve supply and acute and chronic liver injury has previously been suggested [11]. Some studies indicated that CCl_4_-induced acute hepatotoxicity was promoted by the systemic sympathetic tone or adrenoceptor stimulation [12–16], whereas other investigators found inconsistent effects of the sympathetic nervous system (SNS) on the acute hepatotoxicity of CCl_4_ [17–19]. Thus the role of SNS action in CCl_4_ hepatotoxicity remains controversial and detailed mechanisms of SNS effect do not appear to have been sufficiently elucidated.

Moreover, CCl_4_ is a well-known compound for the inducing immune responses and inflammation [20–22]. Immune cells express various adrenergic and purinergic receptors that are sensitive to transmitters of the SNS. The production of cytokines/chemokines is modulated by activation of these receptors [23]. We therefore hypothesized that the sympathetic activity has an effect on secretion of cytokines/chemokines in an animal with CCl_4_ intoxication. Here, we applied chemical sympathectomy to investigate the role of the SNS on (1) acute toxic liver necrosis and steatosis induced by CCl_4_, and (2) systemic pattern of pro- and anti-inflammatory proteins in CCl_4_-poisoned mice.

## Materials and Methods

### Animals

Wild-type C57Bl/6JNarl male mice (8 weeks old) were purchased from the National Laboratory Animal Center (Taipei, Taiwan) and maintained under specific-pathogen-free conditions in the Laboratory Animal Center of National Defense Medical Center (Taipei, Taiwan). The animals were housed in a constant temperature room (22 ± 1°C) and subjected to a 12:12 hr light/dark cycle (lights on at 07:00 AM) with water and food ad libitum. To decrease the stress status and be relaxed and calm during the time of investigation, animals were regularly handled for a week prior to commencing an experiment. The protocols for all experiments involving mice were approved by the Committee on Institutional Animal Care and Use (IACUC-14–123, IACUC-14–311, and IACUC-15–032), National Defense Medical Center.

### Chemical sympathectomy

The peripheral sympathetic nerve terminals of mice were ablated by intraperitoneal injection of a neurotoxin, 100 mg/kg of 6-hydroxydopamine (6-OHDA) (Sigma, St. Louis, MO, USA), for 5 consecutive days. 6-OHDA was freshly prepared in saline containing 0.1% (wt/vol) ascorbic acid (Sigma). Control animals received solvent only. As 6-OHDA does not cross the blood-brain barrier in adult animals, it selectively ablates peripheral sympathetic nerves [24–26]. The regimen of 6-OHDA treatment has been proven to deplete peripheral catecholamine in rodent tissues [17]. Sympathectomy was confirmed by staining fresh frozen sections of liver with an anti-tyrosine hydroxylase (TH) antibody [27].

### Mice model of acute hepatotoxicity

One day after sympathetic denervation, CCl_4_ (Sigma) was administered intraperitoneally using the 100 μL Hamilton syringe at a single dose of 2 mL/kg, 12.5% diluted in olive oil (Sigma) for acute hepatotoxicity model. This dose which represents approximately one-fifth of the lethal dose in C57BL/6 mouse was chosen to ensure hepatocellular necrosis with low or negligible mortality [28,29]. Control mice were given olive oil at the same dose.

### Experimental design

**Fig 1:**
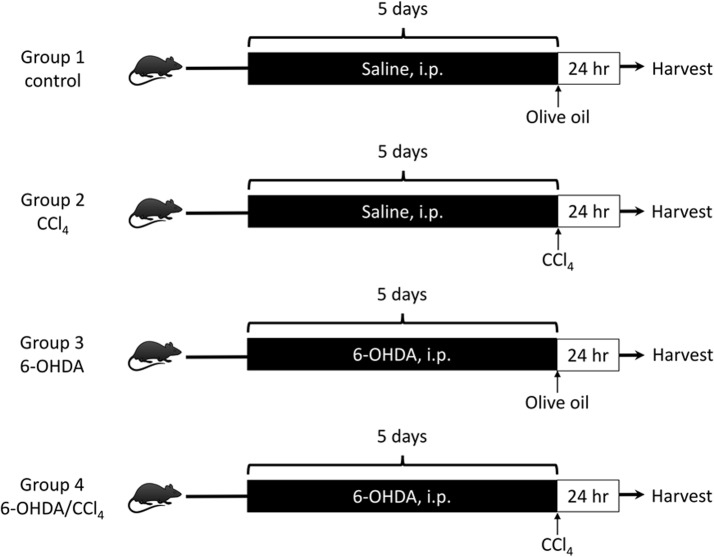
Schematic diagram of the study protocol. Mice were randomly assigned in a blinded manner to one of four groups: group 1, control (n = 6), group 2, CCl_4_ (n = 6), group 3, OHDA (n = 6), and group 4, 6-OHDA/CCl_4_ (n = 6). The animals underwent 5 days of intra-peritoneal (i.p.) administration of 6-OHDA or saline. After ablation of the sympathetic nervous system, the animals were given CCl_4_ or vehicle on day 6, and sacrificed 24 hours after CCl_4_ poisoning. 6-OHDA = 6-hydroxydopamine, CCl_4_ = carbon tetrachloride.

The experimental protocol is described in Fig. 1. Mice were divided into 4 groups (6 mice/group): Group 1 (control), Group 2 (CCl_4_), Group 3 (6-OHDA), and Group 4 (6-OHDA/CCl_4_). After a single dose of CCl_4_ or its vehicle, animals were sacrificed and their livers and blood were harvested for analysis at 24 hours. Livers were divided with lobes then fixed in 10% neutral buffered formalin for histology, preserved in optimum cutting temperature (OCT) (Tissue-Tek, Sakura, CA, USA) compound for frozen sections, and snap-frozen in liquid nitrogen for further protein analysis.

A further set of male C57Bl/6JNarl 8-week old mice was included for additional experiments to establish dexamethasone-mediated effects on the inflammatory response. Dexamethasone (Sigma) was administered by intraperitoneal injection (10 mg/kg), at 15 hours and 2 hours prior to CCl_4_ administration. Two groups of mice (n = 4 for each group) were analyzed in this study: mice pretreated with phosphate-buffered saline (PBS) and subsequently injected with CCl_4_ and mice pretreated with dexamethasone and subsequently injected with CCl_4_. Twenty-four hours later, a blood sample was obtained to confirm liver damage through hepatic enzymes concentrations and a cytokine antibody array.

### Histological and immunofluorescent assay

For histological analysis, formalin-fixed tissues were paraffin-embedded, sectioned (5 μm) and stained with hematoxylin and eosin. Cryostat sections of fresh frozen tissue were stained with Oil-Red-O (Sigma) for lipid detection or fixed in a mixture of methanol and acetone (1:1) at −20°C for 5 min for immunofluorescence. Following blocking of nonspecific activity by incubation with 2% bovine serum albumin (BSA) (Sigma) in TBST (12.5 mM Tris/HCl, pH 7.6, 137 mM NaCl, 0.1% Tween 20) for 20 min, sections were incubated for 1 hr at room temperature with an antibody against TH (Millipore, Billerica, MA, USA), then washed with TBST. Next, they were incubated with donkey anti-rabbit Alexa Fluor 488 (Invitrogen Corporation, Carlsbad, CA, USA) (1:5000 diluted in 2% BSA) for 1 hr at room temperature. Nuclei were counterstained with 4',6-diamidino-2-phenylindole (DAPI) (Sigma). Images were captured using an Olympus microscope BX51 and digital camera DP71 (Olympus, Tokyo, Japan). As described previously [30], the relative areas of centrilobular necrosis (the areas full of vacuolated and necrotic hepatocytes) and steatosis with Oil-Red-O staining were quantitated by ImageJ software (US National Institutes of Health) in six random different fields.

### Measurement of hepatic enzymes and lipid profile

Measurement of serum lactate dehydrogenase (LDH, IU/L), alanine aminotransferase (ALT, IU/L), aspartate aminotransferase (AST, IU/L), alkaline phosphatase (ALP, IU/L), cholesterol (mg/dL), triglycerides (mg/dL), and high-density lipoprotein (HDL, mg/dL), was performed using standard enzymatic procedures by Union Clinical Laboratory (Taipei, Taiwan). Low-density lipoprotein (LDL) and very low-density lipoprotein (VLDL) were estimated by using Freidewald's equation [31].

### Determination of the atherogenic index (AI) and the cardiac risk factor (CRF)

The AI was calculated as (cholesterol-HDL)/HDL. The CRF was calculated as (cholesterol/HDL) [32].

### Liver homogenate preparation and lipid peroxidation assay

Liver samples were lysed using ice-cold standard RIPA buffer (Sigma), 100 μM Na_3_VO_4_ (Sigma), and protease inhibitor cocktail tablet (Roche Diagnostics, Mannheim, Germany). Liver lysates were collected after centrifugation at 13,300 rpm for 15 min. The steady-state level of MDA was analyzed by measuring the formation of thiobarbituric acid reactive substances (TBARS) using a commercial assay kit (Cayman Chemical, Ann Arbor, MI, USA) in accordance with the manufacturer’s protocol. TBARS concentration was calculated from a MDA standard curve and normalized for protein content.

### Measurement of inflammatory cytokines/chemokines in serum

Serum samples were analyzed with a cytokine antibody array, specifically the RayBio Mouse Inflammation Antibody Array 1 (RayBiotech, Inc., Norcross, GA, USA). Briefly, arrays were blocked with blocking buffer, equal amounts of serum were added, and the arrays were incubated at room temperature for 30 minutes. Arrays were processed according to the manufacturer’s instructions. The membranes were scanned by a UVP BioSpectrum AC image system (VisionWorks LS, UVP, Upland, CA, USA). Densitometry was performed by ImageJ software to determine the relative protein expression levels between groups.

### Statistical analysis

Data were expressed as mean ± SD. Differences were analyzed by Student’s t-test for group comparison using the GraphPad Prism software program (GraphPad Software, La Jolla, CA). A *p* value less than 0.05 was considered significant.

## Results

### Identification of the intrahepatic sympathetic nerve fibers

**Fig 2:**
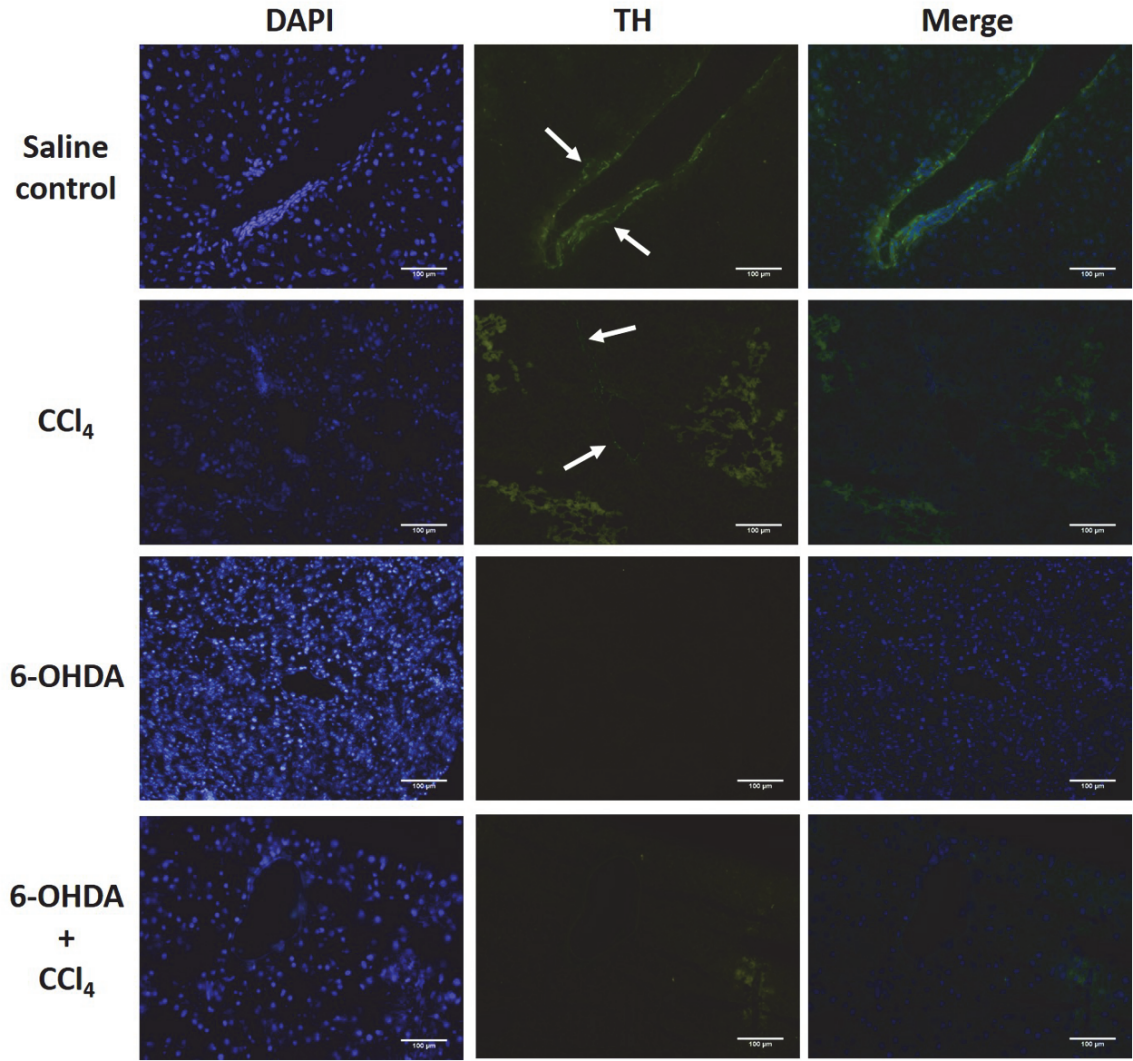
Immunofluorescent analysis of liver tyrosine hydroxylase (TH) reactive nerve fibers. TH positive nerve fibers (arrows) were seen around the portal area in saline treated mice but not in chemical sympathectomised animals. Magnification × 200. The results are representative of four sets of experiments. Scale bar = 100 μm.

The sympathetic nerve marker tyrosine hydroxylase (TH) was used to identify the presence of intrahepatic sympathetic nerve fibers [33]. Immunofluorescences showed that the TH-positive fibers in the liver without chemical sympathectomy were mainly distributed in the portal tract. Chemical denervation by 6-OHDA effectively depleted the TH-positive fibers in the liver (Fig. 2).

### Effect of chemical sympathetic denervation on the CCl_4_-induced acute hepatotoxicity

**Fig 3:**
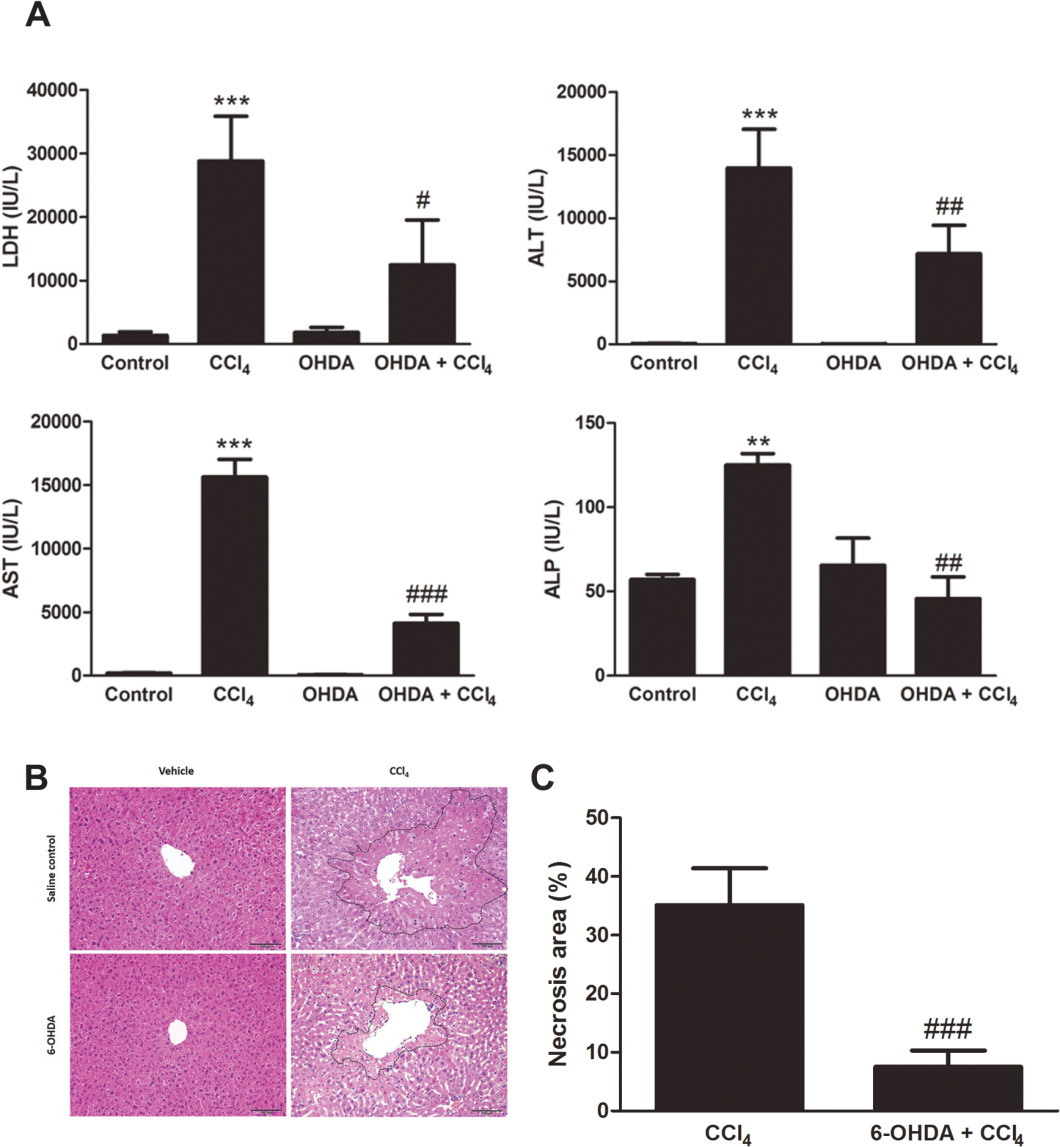
Absence of the sympathetic nervous system attenuated CCl_4_-induced hepatotoxicity. (A) Serum isolated from whole blood was used to determine lactate dehydrogenase (LDH), alanine aminotransferase (ALT), aspartate aminotransferase (AST), and alkaline phosphatase (ALP) levels. Bars are means ± SD, n = 6 mice per group. (***p < 0.001 vs. control, ^#^ *p* < 0.05 vs. CCl_4_, and ^##^ *p* < 0.01 vs. CCl_4_) (B) Liver sections after CCl_4_ administration were stained with hematoxylin and eosin, 200× magnification. Scale bar = 100 μm. (C) The semi-quantitative data of hepatocellular necrosis area showed sympathetic denervation resulted in lower necrosis area (^###^ *p* < 0.001 vs. CCl_4_). Images are representative of n = 6 mice per group.

The biochemical effects of chemical sympathectomy on CCl_4_-induced hepatotoxicity were assayed by assessing serum levels of LDH, ALT, AST, and ALP (Fig. 3A). There was no statistically significant difference between control and 6-OHDA-treated mice. Twenty-four hours after administration of CCl_4_, LDH level was elevated from 1,351 ± 564 to 28,831 ± 7,027 IU/L (*p* = 0.0005); ALT level was elevated from 61 ± 33 to 13,970 ± 3,069 IU/L (*p* = 0.0003); AST level was elevated from 189 ± 70 to 15,620 ± 3,421 IU/L (*p* = 0.0001); and ALP level was elevated from 57 ± 4 to 125 ± 12 IU/L (*p* = 0.0049). Intraperitoneal injection of 6-OHDA inhibited the CCl_4_-induced elevation of serum LDH (12,460 ± 4,072 IU/L, *p* = 0.0465), ALT (7,180 ± 2,240 IU/L, *p* = 0.0040), AST (4,115 ± 1,755 IU/L, *p* < 0.0001), and ALP (46 ± 26 IU/L, *p* = 0.0047) levels (Fig. 1). Histology demonstrated that acute liver necrosis around pericentral areas in the CCl_4_-treated mice (35.19 ± 6.3%) was significantly greater than in the 6-OHDA/CCl_4_-cotreated mice (7.5 ± 2.8%, *p* = 0.0002). 6-OHDA-treated mice showed no morphological necrosis (Fig. 3B and 3C).

### Effect of chemical sympathetic denervation on CCl_4_-induced hepatic lipid accumulation

**Fig 4:**
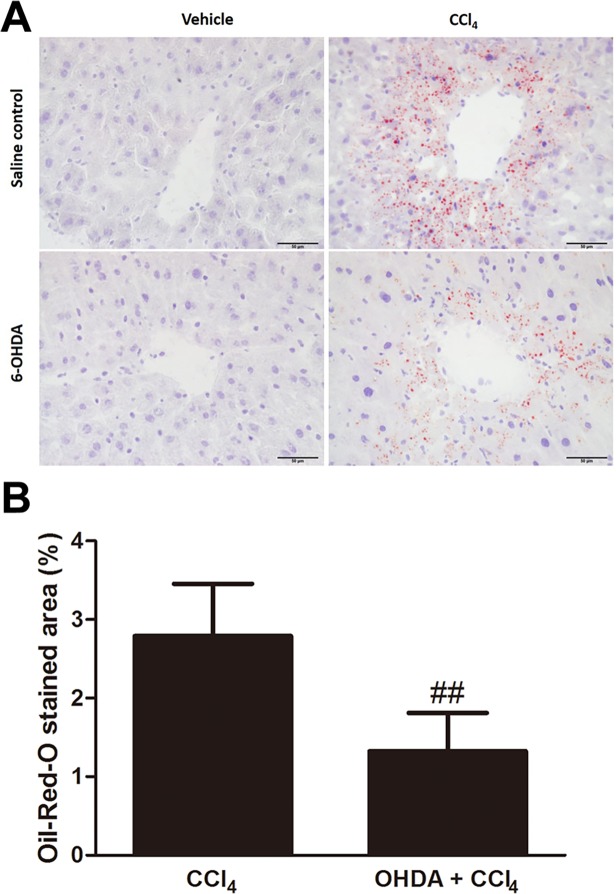
Absence of the sympathetic nervous system attenuated CCl_4_-induced steatosis. (A) Steatosis was evaluated using fat droplets area per 400× field. Scale bar = 50 μm. (B) Semi-quantitatively sympathetic denervation reduced fat droplets deposition prominently in the injured hepatocytes around the central veins compared to control at 24 h. Bars are means ± SD, n = 6 mice per group. (^##^ *p* < 0.01 vs. CCl_4_).

Steatosis was evaluated by assessing the histological area of fat droplets within hepatocytes in Oil-Red-O stained sections (Fig. 4A). There was negligible Oil-Red-O staining in the control mice. In contrast, in CCl_4_-poisoned mice Oil-Red-O lipid droplets were readily identifiable and amounted to 2.8 ± 0.7% of the total area of the liver sections. In the 6-OHDA/CCl_4_-cotreated mice, the lipid-staining area was significantly less (1.3 ± 0.5%, *p* = 0.0014) than that observed in the CCl_4_-treated group (Fig. 4B).

### Effect of chemical sympathetic denervation on CCl_4_-induced lipid profile in serum

**Fig 5:**
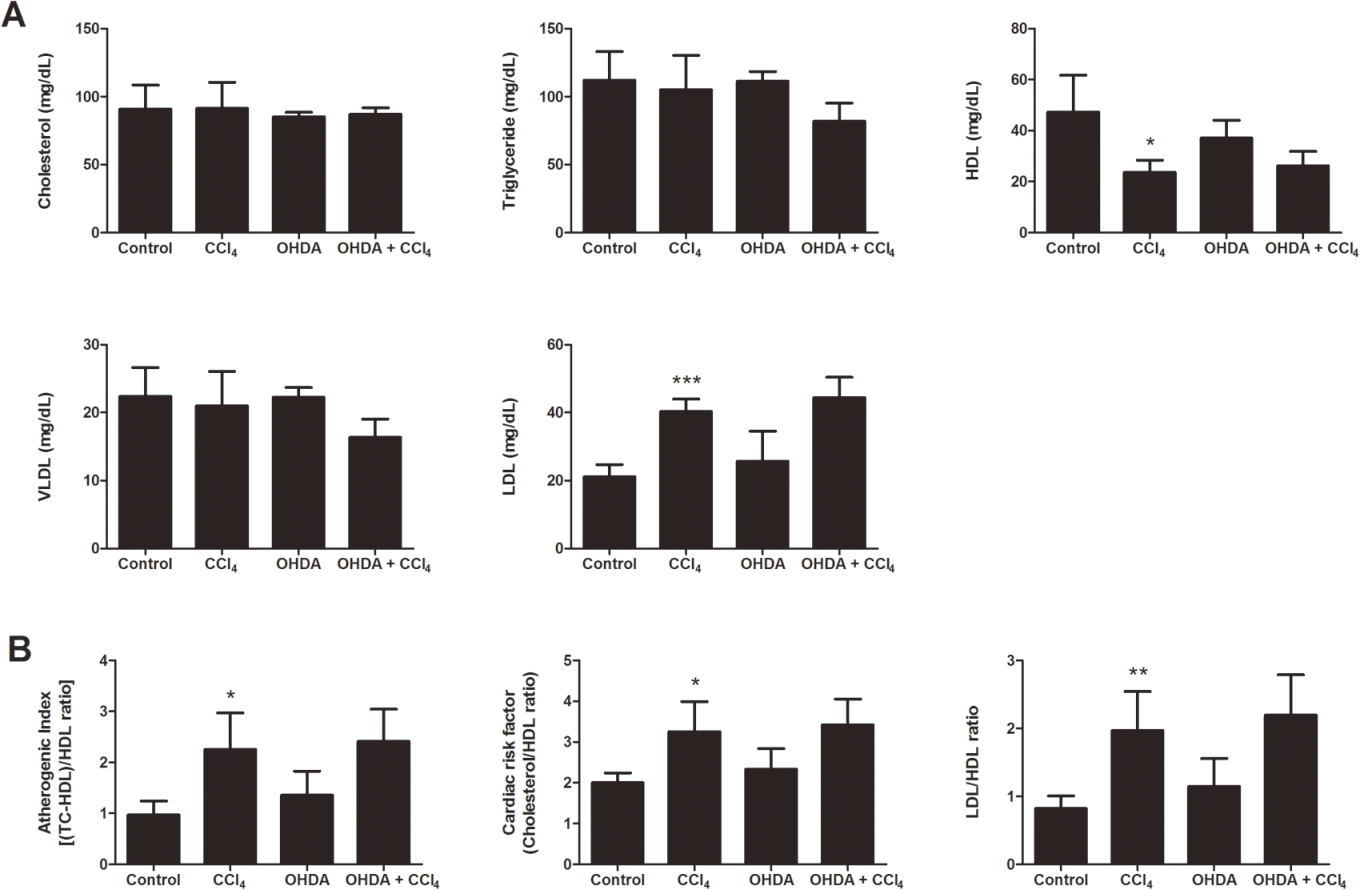
Effect of ablation of the sympathetic nervous system on serum lipid profile in mice. (A) Serum total cholesterol, triglyceride, high-density lipoprotein (HDL), very low-density lipoprotein (VLDL), and low-density lipoprotein (LDL) in mice administered sympathetic denervation for 5 d following CCl_4_ intoxication. (B) Serum atherogenic index, cardiac risk factor, and LDL/HDL ratio in mice with sympathetic denervation following CCl_4_ intoxication. Bars are means ± SD, n = 6 mice per group. In all figures, significant differences are noted as *p < 0.05 vs. control, **p < 0.01 vs. control, and ***p < 0.001 vs. control.

Blood lipid levels are shown in Fig. 5. The levels of cholesterol, triglyceride and VLDL showed no significant difference in the different groups. The levels of HDL (24 ± 5 mg/dL) significantly decreased in CCl_4_-treated mice in comparison to the control mice (47 ± 14 mg/dL, *p* = 0.0448). In contrast, levels of LDL significantly increased in CCl_4_-treated mice (40 ± 4 mg/dL) compared with control mice (21 ± 4 mg/dL, *p* = 0.0003). 6-OHDA/CCl_4_ co-treatment had no effect on these CCl_4_ induced changes in the levels of HDL and LDL. Similarly 6-OHDA/CCl_4_ co-treatment had no influence on the elevated AI index, CRF and LDL/HDL ratio that were seen in the CCl_4_-treated mice.

### Effects of chemical sympathetic denervation on lipid peroxidation levels in liver and serum of CCl_4_-induced liver damage

**Fig 6:**
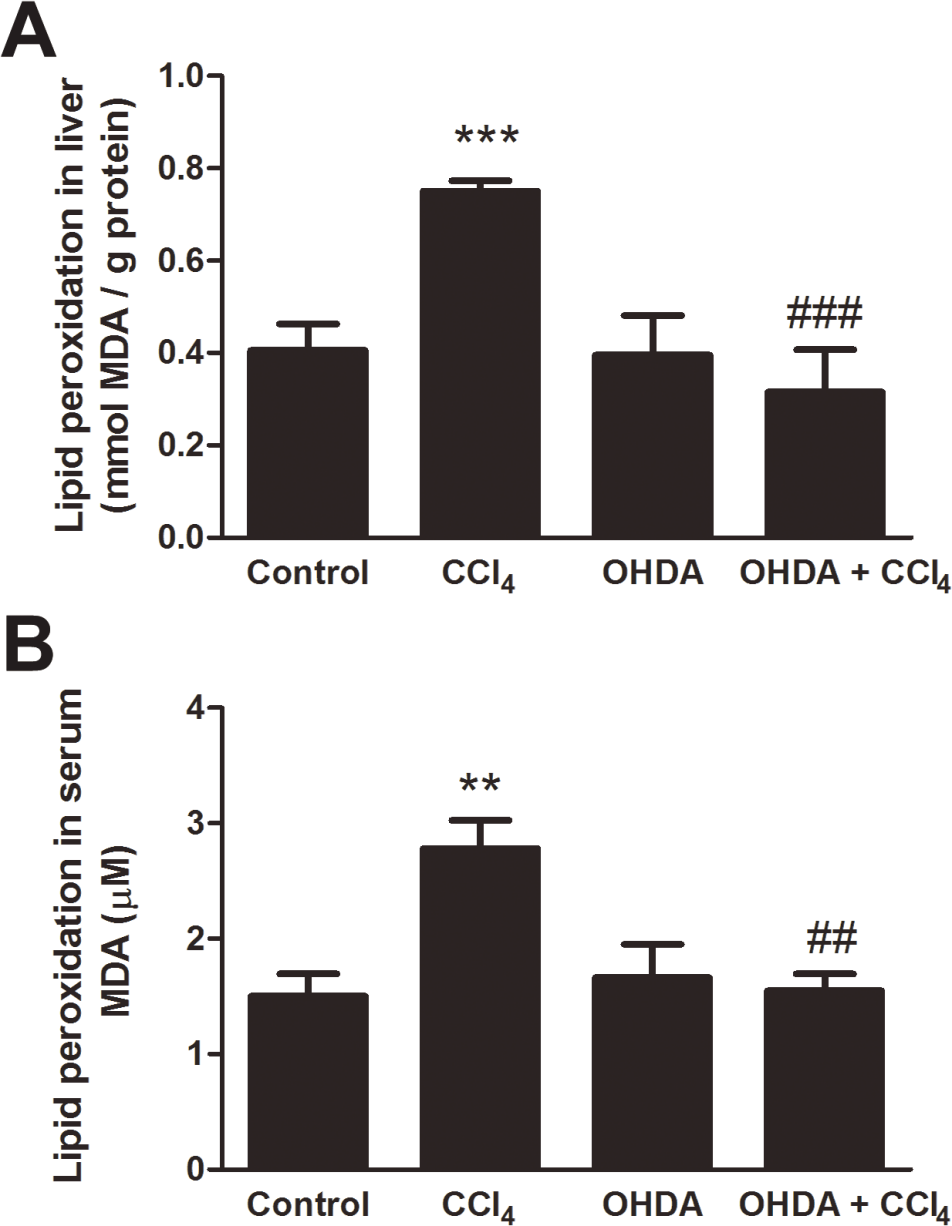
Prevention by sympathetic denervation of CCl_4_-induced lipid peroxidation. Mice were treated or not treated for 24 h with CCl_4_, with or without pretreatment with 6-OHDA. Lipid peroxidation was assessed by malondialdehyde (MDA) measurement in liver homogenates (A) and serum (B). Results are expressed as mmol/g protein in liver homogenates and μM in serum. Data are mean ± SD for 6 mice per group. In all figures, significant differences are noted as **p < 0.01 vs. control, ^##^ *p* < 0.01 vs. CCl_4_, ***p < 0.001 vs. control, and ^###^ *p* < 0.001 vs. CCl_4_.

The lipid peroxidation status in the liver of control and treated mice is shown in Fig. 6A. The level of MDA in liver was increased from 0.41 ± 0.06 mmol/g protein in control mice to 0.75 ± 0.02 mmol/g protein in CCl_4_-treated mice (*p* < 0.0001). 6-OHDA on its own had no significant effect on MDA level (0.40 ± 0.09 mmol/g protein), but the elevation of MDA caused by CCl_4_ was blocked in the mice with pretreated with 6-OHDA (0.32 ± 0.09 mmol/g protein, *p* = 0.0005). Moreover, the level of MDA in serum of CCl_4_-treated mice (2.78 ± 0.24 μM) was significantly higher than that of control mice (1.50 ± 0.19 μM, *p* = 0.0068), 6-OHDA-treated mice (1.66 ± 0.29 μM, *p* = 0.0051), and 6-OHDA/CCl_4_-cotreated mice (1.55 ± 0.15 μM, *p* = 0.0013) (Fig. 6B).

### Effects of chemical sympathetic denervation on inflammatory cytokines/chemokines expression in serum of mice after CCl_4_ exposure

**Fig 7:**
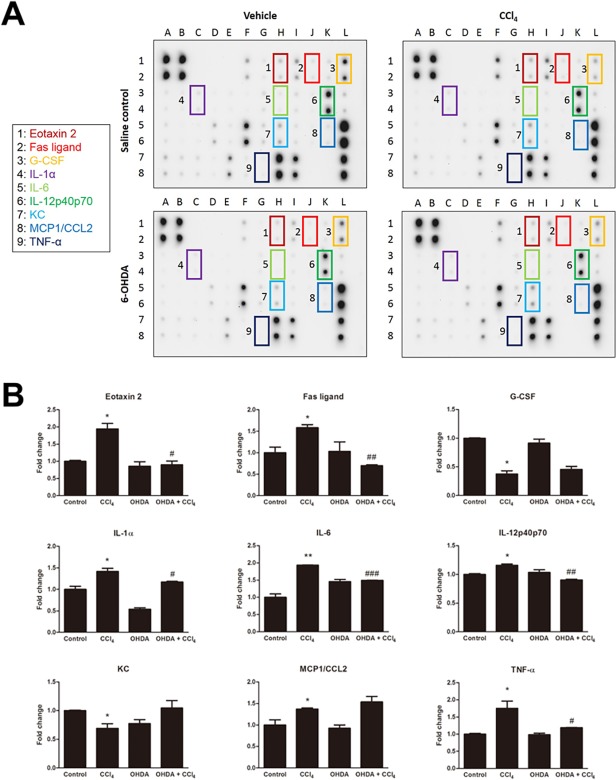
The effect of sympathetic denervation on CCl_4_-induced systemic inflammatory markers as measured by antibody array. (A) Inflammation array in serum from control, CCl_4_-, 6-OHDA-, and 6-OHDA/CCl_4_-treated mice. Altered cytokines and chemokines are indicated by boxes. (B) Quantification of the expression of eotaxin-2/CCL24, Fas ligand, granulocyte colony-stimulating factor (G-CSF), interleukin (IL)-1α, IL-6, IL-12p40p70, keratinocyte-derived chemokine (KC), monocyte chemoattractant protein-1 (MCP-1/CCL2), and tumor necrosis factor-α (TNF-α) in each group. Data are presented as the fold of expression. Bars are mean ± SD for two replicated spots on the membrane. In all figures, significant differences are noted as *p < 0.05 vs. control, ^#^ *p* < 0.05 vs. CCl_4_, **p < 0.01 vs. control, ^##^ *p* < 0.01 vs. CCl_4_, and ^###^ *p* < 0.001 vs. CCl_4_.

Serum from mice in each treatment group was exposed to the inflammatory cytokines/chemokines antibody arrays (Fig. 7A). The levels of eotaxin-2/CCL24, Fas ligand (FasL), interleukin (IL)-1α, IL-6, IL-12p40p70, monocyte chemoattractant protein-1 (MCP-1/CCL2), and tumor necrosis factor-α (TNF-α) each increased in CCl_4_-treated mice group and SNS ablation significantly prevented these cytokines and chemokines except MCP-1. Serum samples obtained from CCl_4_-treated mice expressed decreased levels of the granulocyte colony-stimulating factor (G-CSF) and keratinocyte-derived chemokine (KC) as compared to control mice. Both showed no significant change in 6-OHDA/CCl_4_-cotreated mice (Fig. 7B).

### Effects of pretreatment with dexamethasone on the systemic inflammatory response of mice following CCl_4_ exposure

**Fig 8:**
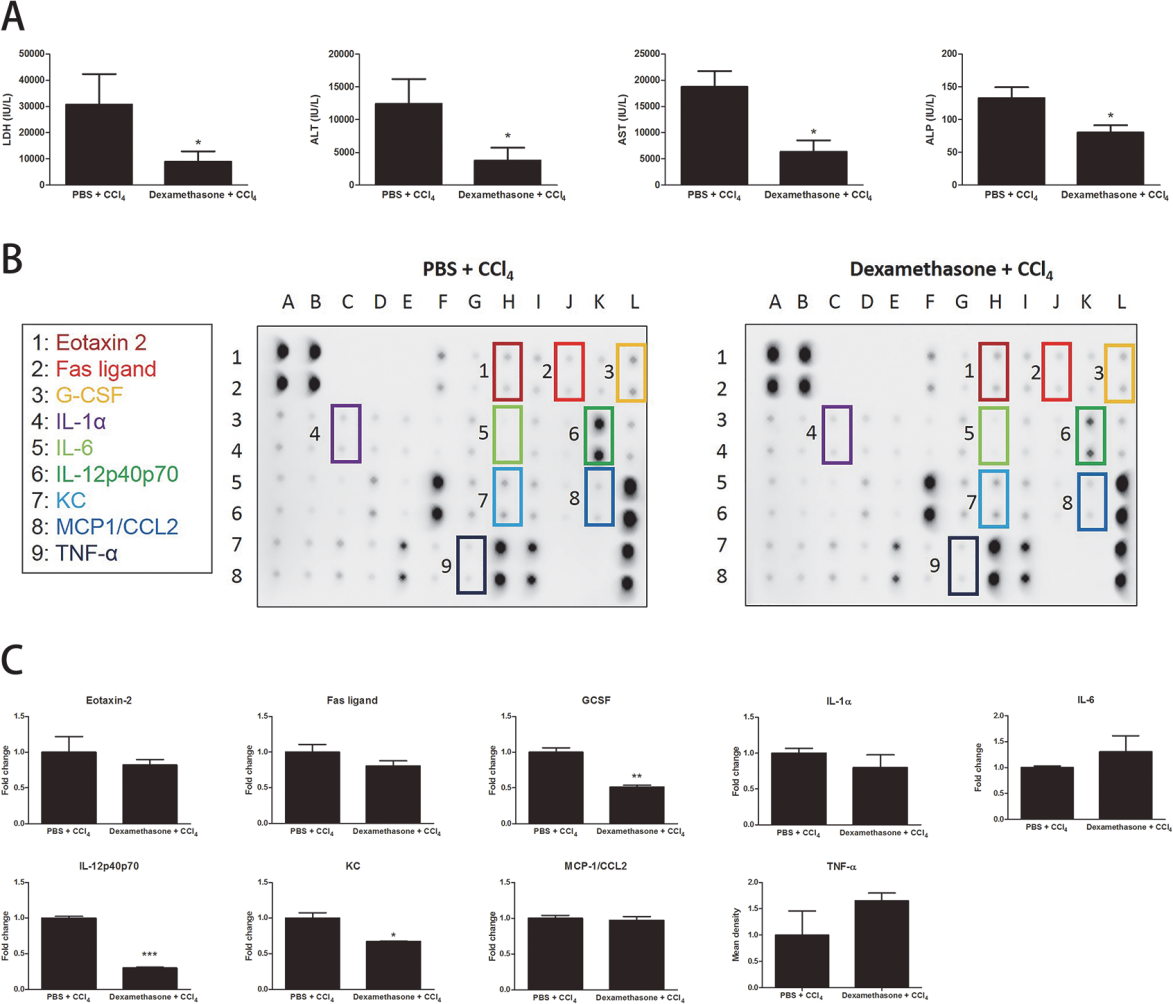
The effect of dexamethasone pretreatment on CCl_4_-induced hepatotoxicity and systemic inflammation. (A) Serum isolated from whole blood was used to determine LDH, ALT, AST, and ALP levels. Bars are means ± SD, n = 4 mice per group. (*p < 0.05 vs. PBS + CCl_4_) (B) Inflammation array in serum from PBS + CCl_4_ and Dexamethasone + CCl_4_-treated mice. (C) Quantification of the expression of eotaxin-2, FasL, G-CSF, IL-1α, IL-6, IL-12p40p70, KC, MCP-1/CCL2, and TNF-α in each group. Data are presented as the fold of expression. Bars are mean ± SD for two replicated spots on the membrane. In all figures, significant differences are noted as *p < 0.05 vs. PBS + CCl_4_, **p < 0.01 vs. PBS + CCl_4_, and ***p < 0.001 vs. PBS + CCl_4_.

The biochemical impacts of dexamethasone pretreatment on CCl_4_-induced hepatotoxicity were assayed by assessing serum levels of LDH, ALT, AST, and ALP (Fig. 8A). Dexamethasone significantly decreased CCl_4_ induced hepatotoxicity. The levels of liver enzymes in the peripheral blood in dexamethasone/CCl_4_ vs. PBS/CCl_4_ treated mice were LDH 8,931 ± 3,957 vs. 30,700 ± 11,610 IU/L (*p* = 0.0371), ALT 3,739 ± 1,963 vs. 12,390 ± 3,795 IU/L (*p* = 0.0247), AST 6,370 ± 2,192 vs. 18,700 ± 3,062 IU/L (*p* = 0.0436), and ALP 80 ± 11 vs. 133 ± 17 IU/L (*p* = 0.0315). Furthermore, serum from mice in PBS/CCl_4_ group and dexamethasone/CCl_4_ group was exposed to the inflammatory cytokines/chemokines antibody arrays (Fig. 8B). Serum samples obtained from dexamethasone/CCl_4_-treated mice expressed decreased levels of the IL-12p40p70 (0.30 ± 0.01 fold; *p* = 0.0007), G-CSF (0.51 ± 0.02 fold; *p* = 0.0101) and KC (0.67 ± 0.00 fold; *p* = 0.0238) as compared to PBS/CCl_4_ group. Others tested showed no significant change (Fig. 8C).

## Discussion

The role of SNS in CCl_4_-induced acute hepatotoxicity is still a matter of debate [12–14,17–19]. It has been shown that selective sympathetic blockade confined to liver could alleviate liver injury by CCl_4_ [12]. Prior chemical sympathectomy with 6-OHDA abolished the CCl_4_ toxicity, suggesting that it is mediated through adrenergic stimulation of the liver, but the regulatory mechanism of sympathetic innervation remains poorly understood so far [13,14]. However, it has been demonstrated that the inhibitory effect of noradrenaline on acute hepatotoxicity induced by CCl_4_ [19]. In addition, it has been reported that pretreatment of a ganglionic blocking agent diminished acute hepatotoxicity of CCl_4_ only in female Wistar rats rather than in males [18]. In contrast, in the pilot experiment conducted by Dubuisson and colleagues, chemical destruction of noradrenergic fibers or antagonism of noradrenergic signaling through α_1_ receptor did not decrease the acute toxicity of CCl_4_, as checked by biological tests and necrosis area measurement [17]. The discrepancy between the previous studies can probably be accounted for by the use of different species, varied chemical sympathectomy, and dissimilar harvest time points. In this report, we found that chemical sympathetic denervation strikingly attenuated CCl_4_-mediated hepatotoxicity and systemic inflammatory cytokines production in mice. These data suggest that SNS enhances hepatotoxicity and immune response in animals primed with a toxin such as CCl_4_.

Hepatic sympathectomy may also be carried out surgically, overcoming potential systemic effects of treatment with 6-OHDA. Surgical sympathectomy is not without its difficulties. It is impossible to guarantee full hepatic denervation, previous reports indicating that that the extent of denervation varies widely between 30 and 78% in the surgically denervated rodent liver [34,35]. Furthermore, in studies aimed at identifying the pathophysiologic effect of selective hepatic vagotomy on the hepatic sympathetic nerve no significant difference in norepinephrine levels between the sham-operated and vagotomized livers are seen [36]. On the other hand, peritoneal 6-OHDA is rapidly distributed by the circulation into tissues, where sympathetic fibers are destroyed as a consequence of its internalization into recycling synaptic vesicles, where it is subsequently oxidized to generate neurotoxic free radicals. As 6-OHDA does not cross the blood-brain barrier in adult animals, it selectively ablates peripheral sympathetic nerves [24–26,37,38]. Importantly, when a selective hepatic sympathectomy is performed, there is only a disconnection of the sympathetic input from the hypothalamus to the liver, rather than the systemic inhibition of the SNS caused by chemical sympathectomy [38,39]. It was first proposed that the massive discharge of the SNS, occasioned by an action of CCl_4_ on the central nervous system, had diminished hepatic blood flow with associated centrilobular hypoxia, which would lead to centrilobular necrosis [2]. Our study, the effect of sympathetic denervation to reduce CCl_4_-induced acute hepatotoxicity, supports this hypothesis. Indeed the effect of adrenergic signaling in the liver and serum with respect to modulation of CCl_4_-induced lipid peroxidation has not yet been addressed. Our study demonstrated that, during CCl_4_ intoxication, the level of MDA was increased in the liver with an associated increase in MDA concentrations in circulation. Chemical sympathetic denervation exhibited protective effects by reducing CCl_4_-mediated lipid peroxidation through decreased production of free radical derivatives, as evidenced by the decreased MDA levels in liver and serum. Thus, these results indicate that a SNS response to CCl_4_ metabolic intoxication promote the systemic and hepatic lipid peroxidation.

Several studies have reported that hepatosteatosis occurs after acute exposure to CCl_4_ albeit by uncertain mechanisms [10,40,41]. First, CCl_4_ might lead to steatosis due to impaired liver mitochondrial β-oxidation which processes hepatic lipid breakdown [42]. In addition, CCl_4_-induced TNF-α synthesis via stimulation of Kupffer cells might contribute to steatosis [10,43]. Furthermore, CCl_4_-induced liver steatosis was shown to be related to cytochrome P450 2E1 expression and activity in vitro [8] and also in vivo [43], respectively. It is also believed that the lipid accumulation, which commences very early, is due to failure of the liver to transport triglyceride-rich LDL into the plasma [44], which arises from the CCl_4_-mediated ER stress with structural disorganization [9]. Moreover, it has been indicated that a decrease in hepatic glutathione concentration in CCl_4_-injected mice is closely related to triglyceride accumulation in the liver [45]. Additionally, it has been suggested that the loss of microsomal triglyceride transfer protein (MTP) might be involved in the development of CCl_4_-hepatosteatosis because CCl_4_ exposure leads to covalent modification of MTP and its degradation by proteasomes [41]. Finally, CCl_4_ poisoning might affect glycoprotein processing and maturation at the level of liver microsomes and Golgi apparatus. CCl_4_ poisoning might then influence the synthesis, maturation, and release of hepatic VLDL, and thereafter cause fat accumulation [46]. The mechanisms whereby the SNS acts on the metabolism of hepatic lipids have not been clearly identified. It is important to note that our study revealed an overall anti-steatosis role in blocking the SNS signaling in the CCl_4_-induced surfeit of fat in the liver. These observations suggest that the SNS acts as a positive regulator of steatosis signaling pathways in CCl_4_-intoxicated liver. It further suggests that hepatosteatosis following CCl_4_ poisoning is not the result of one single event, but rather, the end result of a series of episodes. Therefore, a new project in our laboratory is now in progress to further clarify the influence of chronic sympathetic denervation in drug-induced hepatosteatosis and other forms of fatty liver.

In our study, LDL levels in serum were significantly increased and HDL levels in serum were significantly decreased after CCl_4_ treatment. These results were reanalyzed for determination of the AI, CRF, and LDL/HDL ratio. However, sympathetic denervation was not effective in modifying serum levels of LDL, HDL, AI, CRF, and LDL/HDL ratio after CCl_4_ treatment. These findings indicate that the SNS may not be involved in the CCl_4_-induced alteration of the lipid profile in the circulation. Taken together, the presence of the SNS is considered the critical event in the development of CCl_4_-induced steatosis which results from an imbalance between lipid synthesis and degradation rather than from a consequence of the failure of triglycerides to move as VLDL from liver to the circulation.

In this study, we showed that acute CCl_4_ intoxication increased circulating levels of inflammatory cytokines/chemokines, including eotaxin 2, FasL, IL-1α, IL-6, IL-12, MCP-1, and TNF-α. The source of the increased levels of these cytokines/chemokines is not clear. It is likely to be, at least in part, from a variety of intrahepatic cells including CD11b^+^ Kupffer cells, stellate cells/hepatic myofibroblasts, endothelial cells in addition to peritoneal mesothelial and exudate cells following CCl_4_ administration [21,47–58]. Previous studies in ischemia and reperfusion models have also demonstrated the ability of hepatocytes to produce IL-12 [59]. In steatotic liver, IL-12 is probably derived from Kupffer cells [60] whereas in chronic viral hepatitis, primary biliary cirrhosis, and fulminant hepatitis B, IL-12 is produced primarily by sinusoidal endothelial cells, hepatic stellate cells, bile ducts, and lymphocytes [61]. It has been shown previously that some catecholamines can induce an inflammatory response in hepatocytes, e.g. overproduction of IL-6 [62]. However, in the current study we have not performed in vitro studies on primary hepatocyte cultures challenged with CCl_4_ in the presence or absence of catecholamines that could more accurately identify the hepatocyte as being a primary source of particular inflammatory mediator production. Nevertheless, as chemical sympathectomy with 6-OHDA treatment leads to potential global alterations in physiology; e.g., decrease of arterial blood pressure, increase of mesenteric blood flow and microcirculatory blood flow, and blood flow to immune organs [37,63,64] with a dramatic alteration of cytokine production [24,37,65–67], it is unclear to what extent these peripheral events are involved in the alterations in chemokine/cytokine levels seen in the current study.

Immune cells express various adrenergic and purinergic receptors that are sensitive to transmitters of the SNS. The production of cytokines and chemokines is modulated by activation of these receptors. Notably, the production of TNF-α, IL-6, and IL-12 have been shown to be altered by activation of these receptors [23]. Therefore, we analyzed the effect of sympathetic denervation on the release of cytokines and chemokines in CCl_4_-induced systemic inflammation model. Major hepatotoxic mediators induced by CCl_4_ are IL-6, TNF, and FasL [21,22,29,68]. In our findings, elevated levels of IL-6, TNF-α, and FasL in CCl_4_ group were markedly reduced by ablation of the SNS. These data suggest that the SNS exacerbated CCl_4_-induced hepatotoxicity through the increased level of serum IL-6, TNF-α, and FasL. Previous studies have reported that eotaxin-2/CCL24, IL-1α, IL-12, and TNF-α, respectively, are the important mediators in the steatosis model [60,69–71]. In this study, eotaxin-2/CCL24, IL-1α, IL-12, and TNF-α production in mice treated with CCl_4_ was increased compared with that in control mice, suggesting these cytokines and chemokines play a critical role in CCl_4_-induced steatosis. Additionally, these cytokines and chemokines were significantly reduced by ablation of the SNS. Therefore, we speculate the SNS contributes to the progress of CCl_4_-induced hepatosteatosis via eotaxin-2/CCL24, IL-1α, IL-12, and TNF-α signaling. To investigate whether dexamethasone reproduces the effect of liver sympathetic denervation in CCl_4_ model, in this study, we found that a simple pretreatment regimen with dexamethasone can partially inhibit CCl_4_-induced hepatic injury and inflammatory immune responses. These experiments were preliminary; further experiments are needed to explore the relationship between an attenuation of the inflammatory response by glucocorticoids and a blunted activation of sympathetic signaling.

In conclusion, the SNS is found to be involved in the process of CCl_4_-mediated acute hepatotoxicity and systemic inflammatory responses. This novel finding provides us with important insights into the pathophysiological significance of the SNS in promoting the CCl_4_ poisoning. As the SNS is early and essential in the onset of CCl_4_-induced lipid peroxidation and steatosis, the findings raise the possibility that the SNS may be involved in the free radical biology and the development of other forms of fatty liver. Additionally, these data reinforce the importance of the SNS in mechanisms of chemical- or drug-induced hepatotoxicity with associated-systemic inflammatory responses.
